# Species composition and overall diversity are significantly correlated between the tongue coating and gastric fluid microbiomes in gastritis patients

**DOI:** 10.1186/s12920-022-01209-9

**Published:** 2022-03-17

**Authors:** Jiaxing Cui, Siyu Hou, Bing Liu, Mingran Yang, Lai Wei, Shiyu Du, Shao Li

**Affiliations:** 1 Institute of TCM-X, MOE Key Laboratory of Bioinformatics / Bioinformatics Division, BNRist / Department of Automation, Tsinghua University, Beijing, 100084 China; 2 China Industrial Control Systems Cyber Emergency Response Team, Beijing, 100040 China; 3 Beijing Lotus BioMedical Technology Co., Ltd., Beijing, 102206 China; 4 State Key Laboratory of Ophthalmology, Zhongshan Ophthalmic Center, Sun Yat-Sen University, Guangzhou, 510060 China; 5 China-Japan Friendship Hospital, Beijing, 100029 China; 6 School of Life Sciences and Center for Synthetic and Systems Biology, Tsinghua University, Beijing, 100084 China

**Keywords:** Microbiome, Tongue coating, Gastric fluid, Correlation

## Abstract

**Background:**

In traditional Chinese medicine, it is believed that the “tongue coating is produced by fumigation of stomach gas”, and that tongue coating can reflect the health status of humans, especially stomach health. Therefore, studying the relationship between the microbiome of the tongue coating and the gastric fluid is of great significance for understanding the biological basis of tongue diagnosis.

**Methods:**

This paper detected the microbiomes of the tongue coating and the gastric fluid in 35 gastritis patients using metagenomic sequencing technology, systematically constructed the microbial atlas of tongue coating and gastric juice, and first described the similar characteristics between the two sites.

**Results:**

There was a significant correlation between tongue coating and gastric juice in terms of microbial species composition and overall diversity. In terms of species composition, it was found that the two sites were dominated by five phyla, namely, Actinobacteria, Bacteroidetes, Firmicutes, Fusobacteria and Proteobacteria, and that most of the gastric microbial species could be detected from the patient's own tongue coating. In terms of overall diversity, a significant correlation was found between the alpha diversity of the tongue coating microbiome and the gastric juice microbiome. Furthermore, in terms of abundance, 4 classes, 2 orders, 4 families, 18 genera and 46 species were found to significantly correlate between the tongue coating and the gastric fluid.

**Conclusions:**

The results provide microbiome-based scientific evidence for tongue diagnosis, and offer a new perspective for understanding the biological basis of tongue diagnosis.

## Background

Traditional Chinese medicine (TCM) believes that the “tongue coating is produced by the fumigation of stomach gas”. The tongue coating as the initial part of the digestive tract, can reflect the health status of humans, especially the status of the stomach. Some studies have characterized the correlation between the tongue coating microbiome and the status of the host, especially with regard to stomach disease. Our group has revealed variations in tongue coating microbiomes in gastritis patients with different TCM subtypes by 16S rRNA gene sequencing, while 123 OTUs enriched in cold patients with typical white-greasy tongue coating, and 258 OTUs enriched in hot patients with typical yellow-dense tongue coating [1]. Furthermore, we have demonstrated the overall diversity and species variation of the tongue coating microbiome during the occurrence and development of gastritis by metagenomic sequencing [2]. Ye’s group found that *Bacillus* only exists in chronic erosive gastritis patients with yellow tongue coatings [3]. Sun and his colleagues used the 16S rRNA denaturing gradient gel electrophoresis method to compare the tongue coating microbes of gastritis patients and healthy people and found that 8 strips were significantly different between the two groups [4]. Han et al. found that the tongue coating of patients with colon cancer was significantly thicker than that of healthy people, and 16S rDNA sequencing showed that there were differences in the tongue coating microbiome between colon cancer patients and healthy people [5].

The gastric microbiome has also been repeatedly shown to be associated with the health of the stomach. Chang Soo Eun et al. revealed a significant difference in the microbial composition of the gastric mucosa between patients with chronic gastritis, intestinal metaplasia and gastric cancer through 16S sequencing [6]. S. Sjostedt et al. found that patients with gastritis, gastric cancer, and a history of gastrectomy had more microbes in the stomach than patients with gastric or duodenal ulcers [7]. Paola Mattarelli et al. studied the colonization of Bifidobacterium in the stomach and found that the Bifidobacteriaceae family was more likely to colonize a stomach containing too little stomach acid, which could result from omeprazole treatment [8]. Erik C. von Rosenvinge et al. used 16S rDNA sequencing to study the changes in the microbiome in gastric juice under different conditions and found that the microbial diversity in gastric juice decreased after antibiotic treatment and organ transplantation, and in HIV/AIDS patients with low immunity or in patients with a gastric juice pH > 4. Immunization was found to be accompanied by a decrease in abundance of *Prevotella, Fusobacterium,* and *Leptotrichia*, and an increase in abundance of *Lactobacillus*. Through transcriptional activity analysis, it was found that the activity of Actinobacteria was decreased in gastric juice, and Campylobacter activity was increased in gastric juice [9]. Francisco Aviles-Jimenez used the microarray G3 PhyloChip method to compare gastric mucosal microbes in non-atrophic gastritis, intestinal metaplasia and intestinal type gastric cancer, and found that bacterial diversity was the lowest in intestinal-type gastric cancer patients, followed by patients with intestinal metaplasia and non-atrophic gastritis. It was also found that the non-atrophic gastritis and intestinal-type gastric cancer groups could be clearly separated by the gastric mucosa microbiome, while the intestinal metaplasia group overlapped with both groups [10]. Olabisi Oluwabukola Coker et al. used 16S rRNA gene sequencing to study the changes of gastric mucosal microbes during the development from superficial gastritis to gastric cancer and found that gastric mucosal microbes were dysregulated in intestinal metaplasia and gastric cancer patients [11]. Rui M Ferreira also found a microbial disorder in the stomach of patients with gastric cancer compared with chronic gastritis patients [12].

Although the tongue coating and gastric fluid microbiome each have a strong correlation with the health status of the stomach, the association analysis of the microbiome between the two sites is still deficient, especially in terms of an in-depth systematic analysis and similar characteristics. This paper constructed the microbial map of the tongue coating and gastric juice and first established the systematic association between two sites through metagenomic sequencing, taking gastritis patients as an example.

## Results

### Sample information

**Fig. 1:**
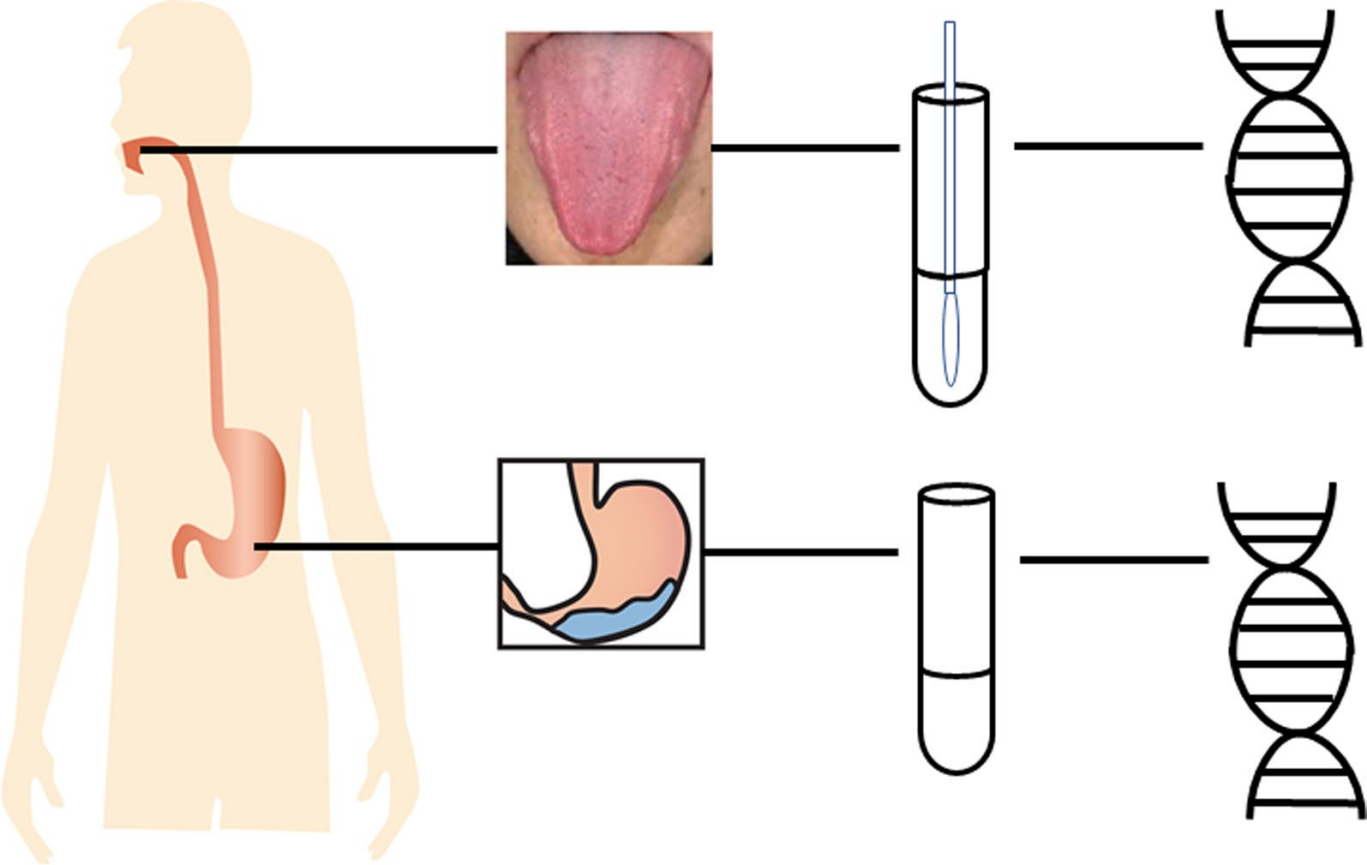
Experimental process of this work

Gastritis patients, including 16 with superficial gastritis, 7 with atrophic gastritis, 4 with intestinal metaplasia and 8 with dysplasia were recruited for this study (Table 1). Tongue coating and gastric fluid samples were collected for all patients. Metagenomic sequencing was conducted to describe the microbiome at these two sites (Fig. 1).

**Table 1:** Sample information

	All samples	Stage			
		Superficial gastritis	Atrophic gastritis	Intestinal metaplasia	Dysplasia
Sample size	35	16	7	4	8
Age (mean ± SD)	56.0 ± 11.4	53.6 ± 13.8	54.1 ± 9.4	58.2 ± 6.8	61.2 ± 9.0
Gender (male/female)	18/17	8/8	4/3	0/4	6/2
BMI (mean ± SD)	24.8 ± 3.8	24.0 ± 4.3	27.3 ± 3.6	24.0 ± 1.9	24.6 ± 3.4
Helicobacter pylori (positive/negative)	14/21	4/12	4/3	3/1	3/5

### Characteristics of the tongue coating microbiome

**Fig. 2:**
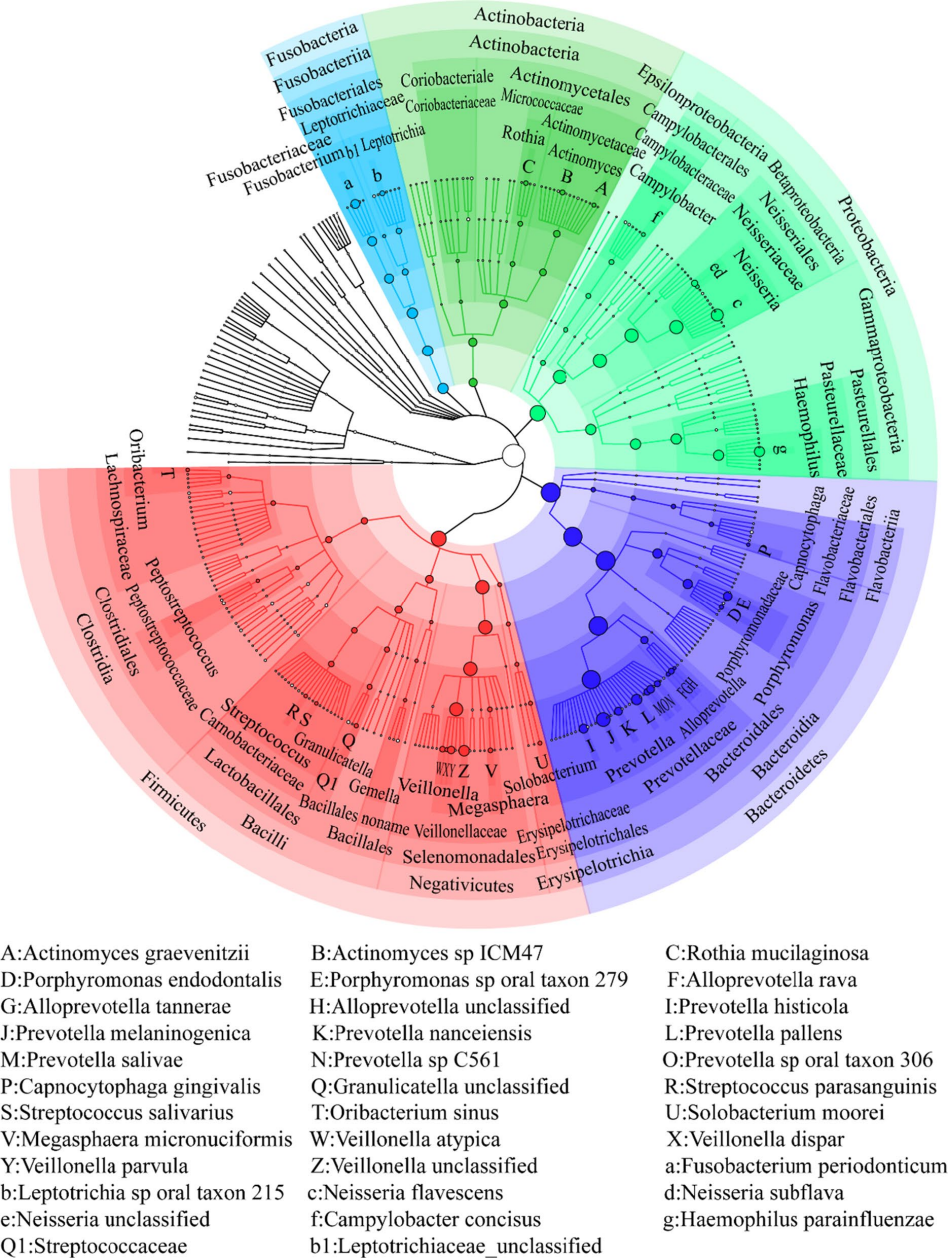
Cladogram of the tongue coating microbiome. From inside to outside are kingdom, phylum, class, order, family, genus, and species. The top 100 most abundant microbes are annotated. Nodes with the same color belong to the same phylum

Tongue coating samples generated 31,672,956–67,989,762 reads per sample. After data analysis, bacteria, viruses and archaea were detected in the tongue coating samples. Bacteria existed in all samples and were the most abundant microbe, with abundances of 91.74–100%. Viruses existed in 30 samples with abundances of 0.00067–8.26%. Archaea were detected in 11 samples with abundances of 0.00065–0.0024%. We constructed a cladogram of the tongue coating microbiome (Fig. 2), and 13 phyla, 32 classes, 36 orders, 68 families, 127 genera and 314 species of bacteria were found in the tongue coating samples.

**Fig. 3:**
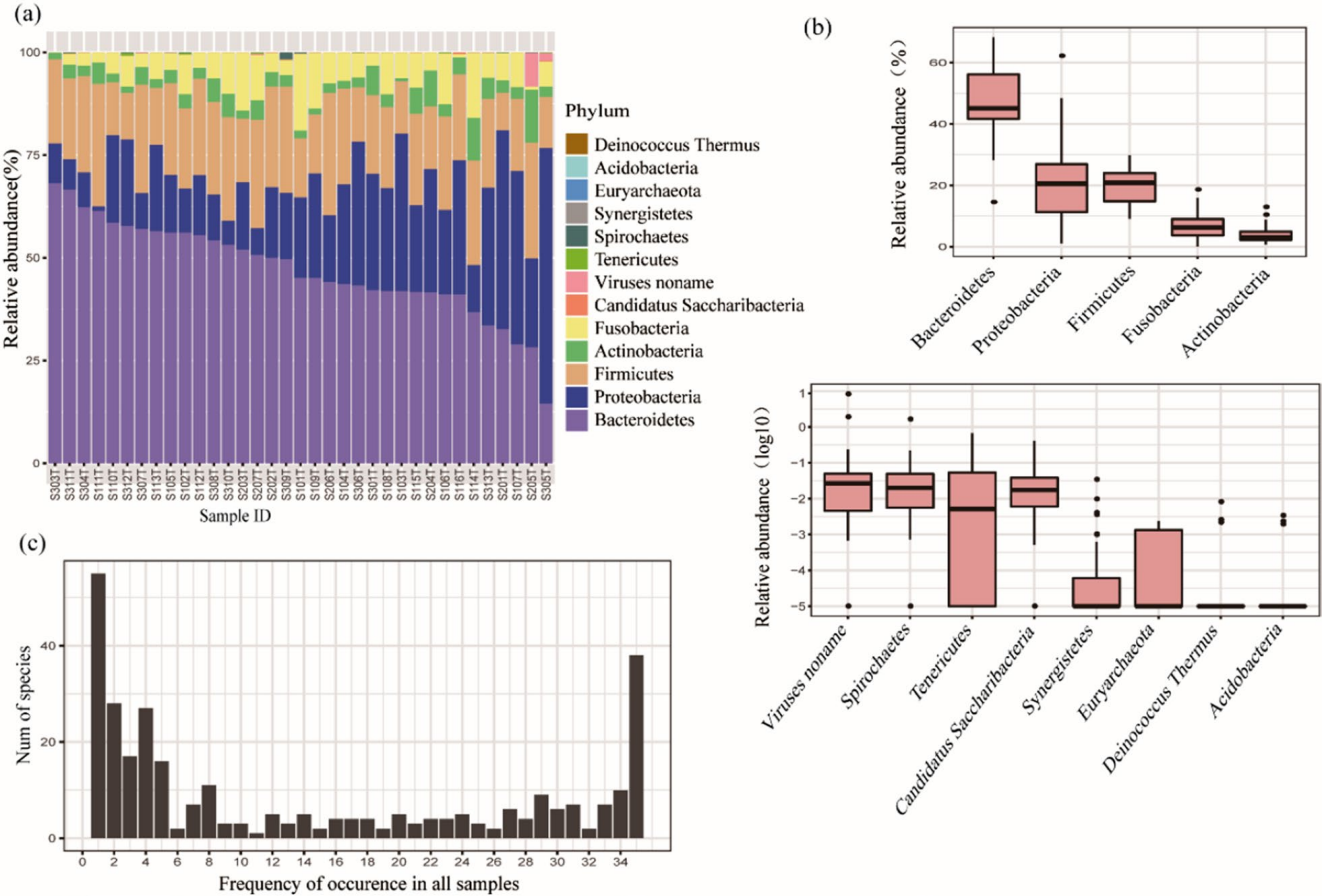
Tongue coating microbiome in all samples. **a** Microbiome constitution in all samples at the phylum level. **b** Boxplot of the tongue coating microbiome at the phylum level. **c** Distribution frequency statistics of species in the tongue coating samples

The most abundant phylum found in the tongue coating samples was Bacteroidetes, followed by Proteobacteria, Firmicutes, Fusobacteria and Actinobacteria (Fig. 3a). The abundance of Proteobacteria differed greatly between different samples, while the abundances of the other four phyla differed slightly between different samples (top). The average abundances of other phyla were less than 1%, and shown as log10 of relative abundance (down) (Fig. 3b). Comparing the species distribution in different samples, 55 species existed only in one tongue coating sample, 38 species existed in all 35 tongue coating samples, and 121 species were present in more than half of the samples, indicating that the distributions of the microbiome were consistent in different tongue coating samples (Fig. 3c).

By annotating the tongue coating microbiome genes using UniRef90, 497127 genes were found. Genes were annotated using the KEGG (Kyoto Encyclopedia of Genes and Genomes) [13–15] database to obtain functions; 4288 KEGG Orthologs (Kos) were found, and the 10 most abundant KOs were K03088, K02358, K02914, K06142, K02078, K03530, K02874, K02904, K02919, and K0296. A total of 357 tongue coating microbiome pathways were obtained when annotated using the MetaCyc database, and the 10 most abundant pathways were PWY-7219, PWY-6700, PWY-6387, PEPTIDOGLYCANSYN-PWY, PWY-6386, PWY-7221, PWY-5686, PWY-7229, PWY-6147, PWY-2942.

### Characteristics of the gastric fluid microbiome

**Fig. 4:**
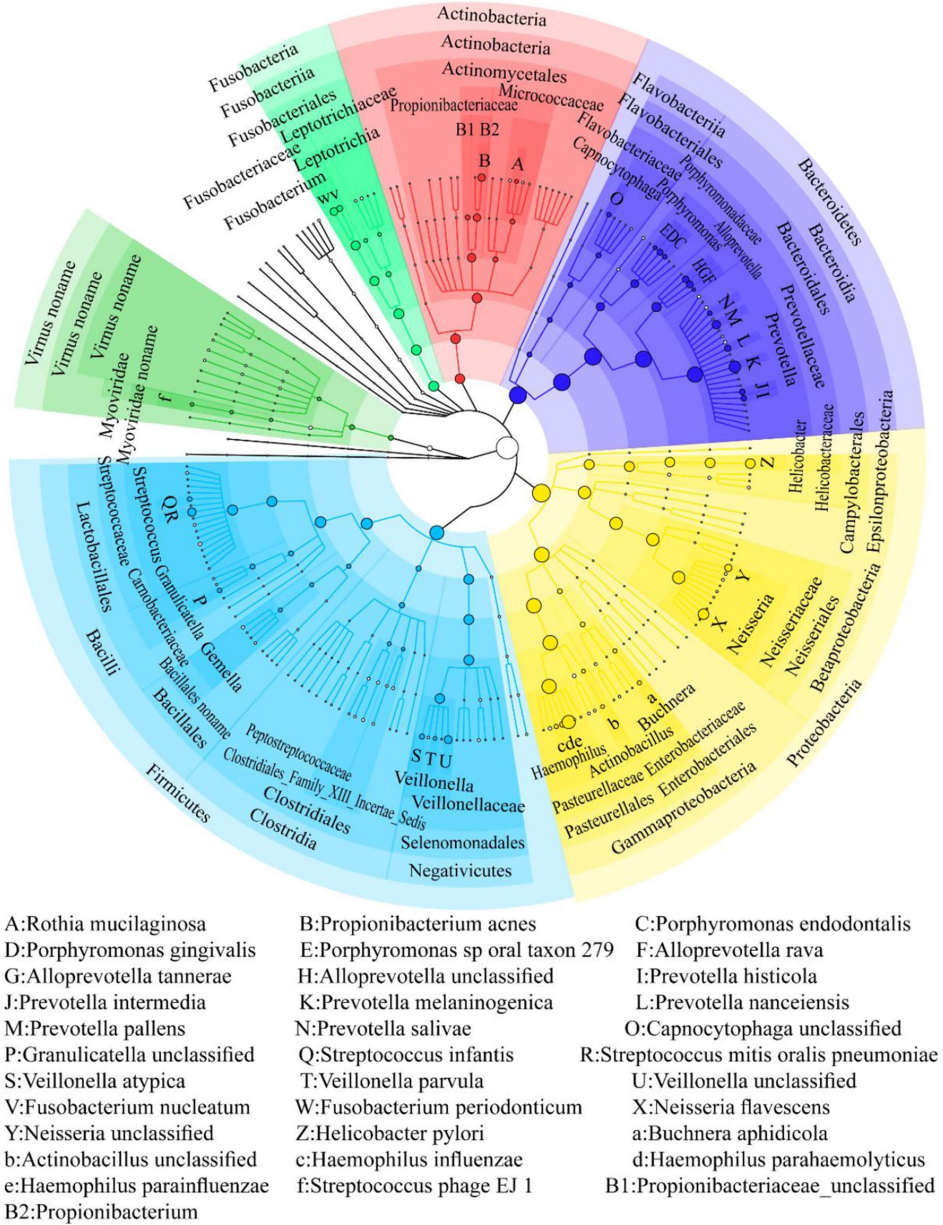
Cladogram of the gastric fluid microbiome. From inside to outside are kingdom, phylum, class, order, family, genus, and species. The top 100 most abundant microbes are annotated. Nodes with the same color belong to the same phylum

Gastric fluid samples generated 13,573,094–38,965,184 reads after metagenomic sequencing. After data analysis, bacteria, viruses, archaea and eukaryotes were found in the gastric fluid samples. Bacteria were found in all gastric fluid samples and were the most abundant microbes with abundances of 83.89–100% in all samples. Viruses existed in 13 samples, with abundances of 0.14–16.11%. Archaea existed in 1 sample with an abundance of 0.082%. Eukaryotes existed in 1 sample with an abundance of 0.35%. Finally, 13 phyla, 21 classes, 31 orders, 58 families, 88 genera, and 194 species of bacteria were detected in the gastric fluid samples. The cladogram of the gastric fluid microbiome is constructed in Fig. 4. The gastric fluid microbiome was dominated by Actinobacteria, Bacteroidetes, Firmicutes, Fusobacteria, and Proteobacteria, which is consistent with previous research by Erik C. von Rosenvinge [9].

**Fig. 5:**
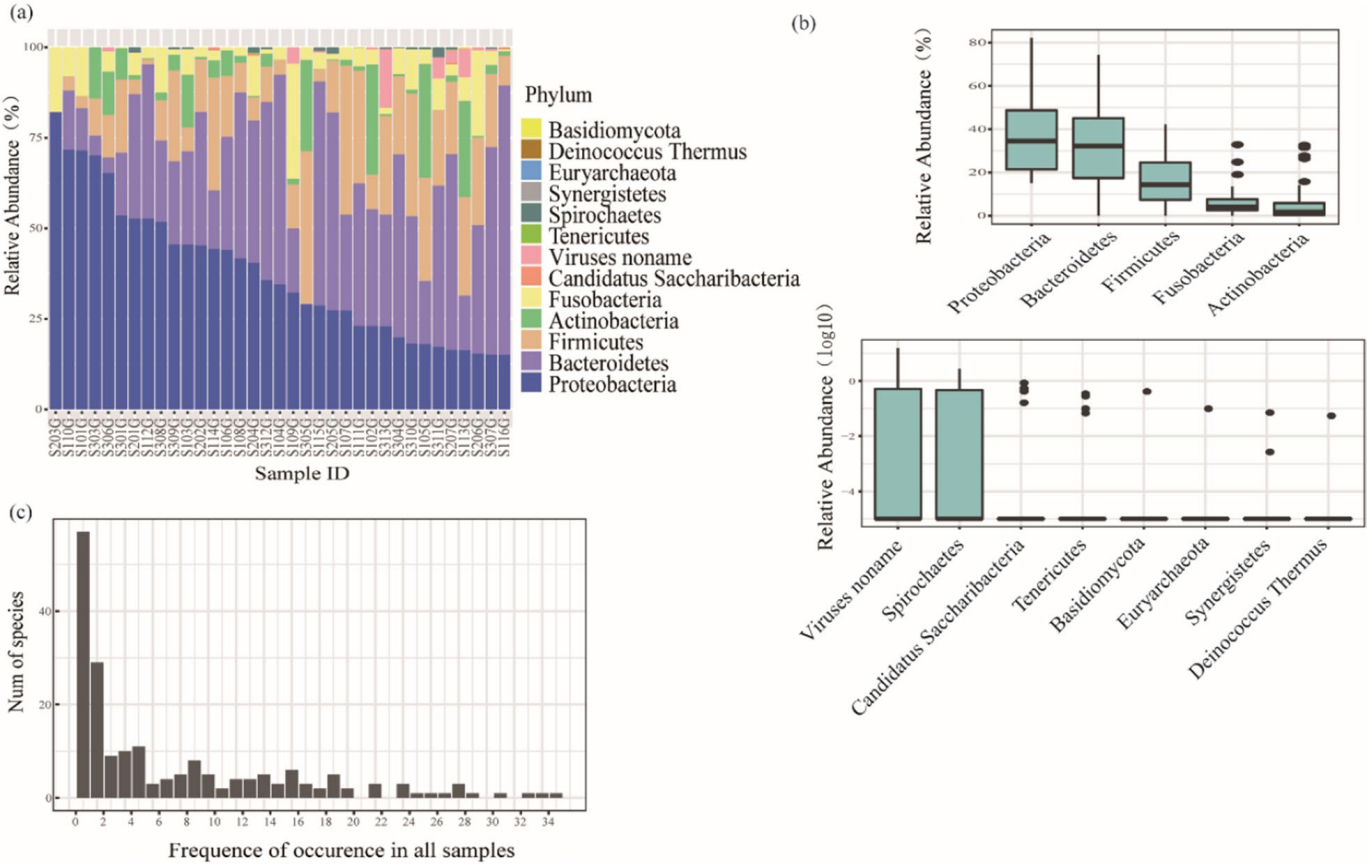
The gastric fluid microbiome in all samples. **a** Microbiome constitution in all samples at the phylum level. **b** Boxplot of the gastric fluid microbiome at the phylum level. **c** Distribution frequency statistics of species in the gastric fluid samples

Proteobacteria and Bacteroidetes were the most abundant phyla in the gastric fluid samples, followed by Firmicutes, Fusobacteria, and Actinobacteria (Fig. 5a). The abundance of Proteobacteria and Bacteroidetes varied greatly between different samples, while the abundance of the other phyla varied little between different samples (Fig. 5b). When comparing the bacterial species distribution in different samples, only *Haemophilus parainfluenzae* existed in all 35 gastric fluid samples, while 57 species were present in only one gastric fluid sample. Twenty-six species were present in more than half of the gastric fluid samples, and most species existed in less than 5 gastric fluid samples (Fig. 5c), which indicated that the distributions of the microbiome were distinctly different in the different gastric fluid samples.

By annotating the gastric fluid microbiome genes using UniRef90, 241136 genes were found. Genes were annotated using the KEGG database to obtain functions; 4248 KOs were found, and the 10 most abundant KOs were K01990, K02358, K03088, K01992, K02078, K06147, K02874, K02015, K02950, and K02948. A total of 309 gastric fluid microbiome pathways were obtained when annotated using the MetaCyc database, and the 10 most abundant pathways were PWY-7219, PWY-7221, PWY-6700, PWY-7229, PEPTIDOGLYCANSYN-PWY, PWY-6386, PWY-6387, PWY-5695, PWY-6126, and PWY-6151.

### Correlation between the tongue coating microbiome and the gastric fluid microbiome

#### Correlation of species construction

**Fig. 6:**
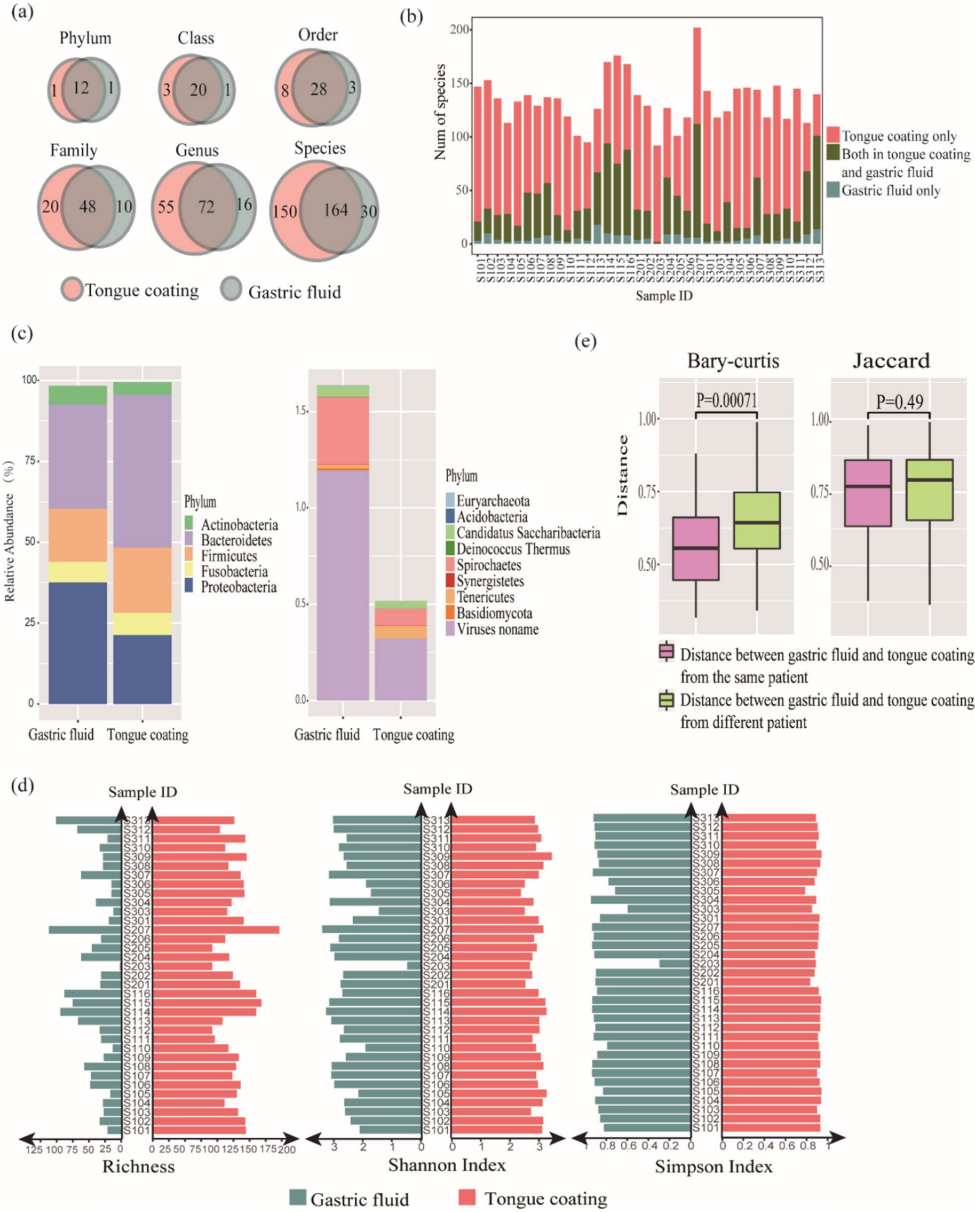
Species construction of the tongue coating and gastric fluid. **a** Venn diagram of the tongue coating and gastric fluid microbiomes. **b** Species consistency between the tongue coating and gastric fluid microbiomes per patient. Each column represents a patient. Green indicates the number of species that can be detected in the patient's gastric juice and tongue coating, red indicates the number of species that can only be detected in the patient's tongue coating, and blue indicates the number of species that can only be detected in the patient's gastric juice. **c** Average abundance of abundant and rare phyla in the tongue coating and gastric fluid. Left, abundant phyla. Right, rare phyla. **d** Alpha diversity of the tongue coating and gastric fluid microbiomes. Each row represents a patient. The green column indicates the alpha index of the gastric fluid microbiome, and the red column indicates the alpha index of the tongue coating microbiome. **e** Beta diversity of the tongue coating and gastric fluid microbiomes from the same patient and from different patients

From the perspective of species presence in the tongue coating and the gastric fluid, there were 12 phyla, 20 classes, 28 orders, 48 families, 72 genera and 164 species present in both the tongue coating and gastric fluid samples. Most microbes in the gastric fluid could be detected in the tongue coating (Fig. 6a). At the phylum level, 12 phyla, namely, Euryarchaeota, Actinobacteria, Bacteroidetes, Candidatus Saccharibacteria, Deinococcus Thermus, Firmicutes, Fusobacteria, Proteobacteria, Spirochaetes, Synergistetes, Tenericutes, and non-name Viruses could be found in both the tongue coating and the gastric fluid, while Basidiomycota was detected only in the gastric fluid, and Acidobacteria was detected only in the tongue coating. For the species present in the tongue coating and the gastric fluid of the same patient, 66.7–100% of gastric fluid species could be found in the patient’s tongue coating (Fig. 6b).

From the perspective of species abundance in the tongue coating and gastric fluid, both dominated by Actinobacteria, Bacteroidetes, Firmicutes, Fusobacteria, and Proteobacteria at the phylum level, each with an abundance of more than 1%. We defined the above five phyla as abundant phyla, while we defined the other nine phyla that were identified in the samples as rare phyla (Fig. 6c).

#### Correlation of overall diversity

We analyzed the overall diversity correlation between tongue coating and gastric fluid from the perspective of alpha and beta diversity. Alpha diversity was characterized by richness, Shannon index and Simpson index in tongue coating and gastric fluid of each patient. Pearson correlation was used to test the significance of the correlation of alpha diversity between the tongue coating and gastric fluid. The richness (P = 0.008), Shannon index (P = 0.012) and Simpson index (P = 0.048) were significantly correlated between the tongue coating and the gastric fluid in the same patient (Fig. 6d), which indicates that species number and distribution were both related in the tongue coating and gastric fluid of the same patient.

Beta diversity was computed based on Bray–Curtis distance and Jaccard distance. The distances between the tongue coating microbiome samples and the gastric fluid samples from the same patient were significantly smaller than those from different patients based on the Bray–Curtis distance after the Wilcoxon rank-sum test (P = 0.0007) (Fig. 6e), which indicated that the tongue coating microbiome was more similar to one’s own gastric fluid microbiome than to the gastric fluid microbiome of others. No significant difference was found based on Jaccard distance after the Wilcoxon rank-sum test, indicating that the tongue coating and gastric fluid correlated not only with the presence of species but also with the abundance of species.

#### Species with a strong correlation between tongue coating and gastric fluid

**Fig. 7:**
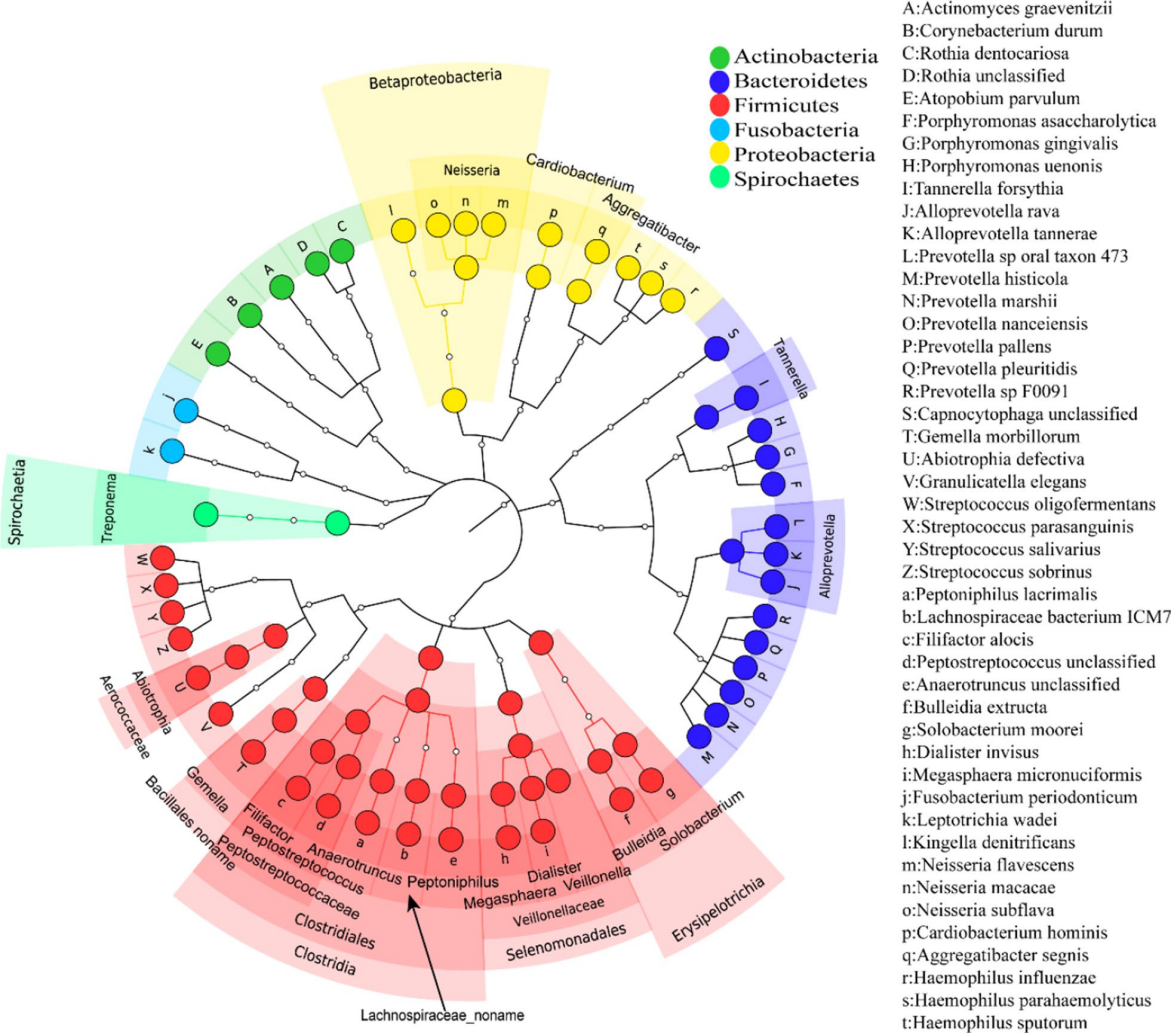
Cladogram of correlated microbiomes. From inside to outside are kingdom, phylum, class, order, family, genus, and species. Correlated microbes are annotated. Nodes with the same color belong to the same phylum

The Spearman correlation test was used to analyze the microbes that were significantly correlated between the tongue coating and the gastric fluid; after false discovery rate correction, 4 classes, 2 orders, 4 families, 18 genera and 46 species were found to have a strong correlation between the gastric fluid and the tongue coating (P < 0.05). Associated microbes were plotted according to taxonomic classification (Fig. 7), and associated microbes were mostly concentrated in 6 phyla, namely Actinobacteria, Bacteroidetes, Firmicutes, Fusobacteria, Proteobacteria, and Spirochaetes.

#### Correlation of gene function

After comparing genes and pathways, in top 10 most abundant genes and pathways, 4 KOs, namely, K02078, K02358, K02874, K03088, and 7 pathways, namely, PWY-6386, PWY-6387, PWY-6700, PWY-7219, PWY-7221, PWY-7229, PEPTIDOGLYCANSYN-PWY were found both in tongue coating and gastric fluid. These discoveries indicated that there was an overall consistency of genes and pathways in the tongue coating and gastric fluid samples.

#### *Helicobacter pylori* in the tongue coating and gastric fluid microbiomes

**Fig. 8:**
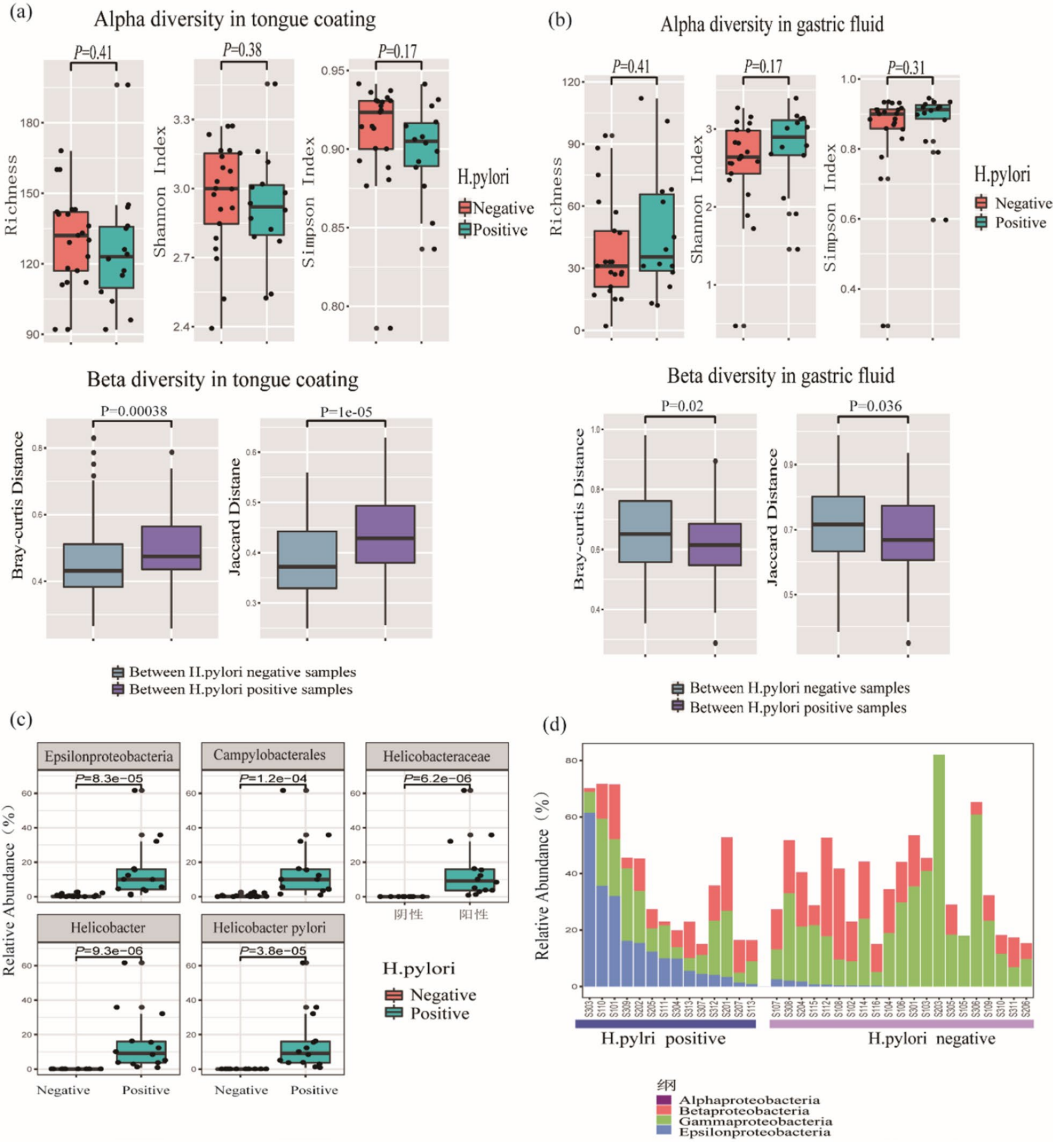
Helicobacter pylori in the tongue coating and gastric fluid microbiome. **a** Top, alpha diversity of HP-positive and HP-negative patients in the tongue coating microbiome. Down, Beta diversity between HP-positive patients and between HP-negative patients in the tongue coating microbiome. **b** Top, alpha diversity of HP-positive and HP-negative patients in the gastric fluid microbiome. Down, beta diversity between HP-positive patients and between HP-negative patients in the gastric fluid microbiome. **c** Differential microbes in the gastric fluid between HP-positive patients and HP-negative patients. **d** The abundances of classes belonging to Proteobacteria in the gastric fluid

As a known microbe related to gastritis and gastric cancer, *Helicobacter pylori* could be detected in the gastric fluid samples of patients whose pathological results for *H. pylori* (*Helicobacter pylori*) were positive, and could not be detected in gastric fluid samples of patients whose pathological results for *H. pylori* were negative; *H. pylori* was not found in the tongue coating of any patients. The tongue coating microbiomes of HP (*Helicobacter pylori*)-positive and HP-negative patients were compared, and alpha diversity was not significantly different between the two groups. For beta diversity, the Bray–Curtis distance and Jaccard distance between HP-positive patients were both higher than those between HP-negative patients (Wilcoxon rank-sum test, P < 0.05) (Fig. 8a), which indicated that there was a larger difference between the tongue coating microbiome of HP-positive patients. After the Wilcoxon rank-sum test followed by FDR correction, no species was significantly different between the HP-positive and HP-negative groups.

For gastric fluid microbes, in terms of alpha diversity, the richness, Shannon index and Simpson index were not significantly different between the HP-positive and HP-negative groups (P > 0.05, Wilcoxon rank-sum test). For beta diversity, Bray–Curtis distances and Jaccard distances between HP-negative patients were significantly higher than those between HP-positive patients (P < 0.05, Wilcoxon rank-sum test) (Fig. 8b). There were smaller differences in the gastric juice microbiomes of HP-positive samples.

After comparing the gastric fluid microbiomes of HP-positive and HP-negative patients by Wilcoxon rank-sum test followed by FDR correction, the abundances of *Epsilonproteobacteria*, *Campylobacterales*, *Helicobacteraceae*, *Helicobacter and Helicobacter pylori* were found to be significantly different between the two groups (P < 0.05, Fig. 8c). The abundance of Proteobacteria, which is the phylum that *H. pylori* belongs to, was not significantly different between the two groups. Comparing all classes belonging to Proteobacteria, we found that the abundance of Epsilonproteobacteria in HP-positive patients was significantly higher than that in HP-negative patients, while the abundance of Betaproteobacteria and Gammaproteobacteria were lower than in HP-negative patients, although the difference was not significant (Fig. 8d). This may also explain why some people with not H. pylori infection are diagnosed with gastritis, while in some cases gastritis patients with H. pylori infection are not cured by eliminating *H. pylori*. We speculate that microbes belonging to the same phylum as *Helicobacter pylori* played a similar role to *H. pylori* in gastritis patients.

## Methods

This research recruited 35 patients who underwent gastroscopy examination in China-Japan Friendship Hospital and were diagnosed with chronic gastritis by gastroscopy and pathological results. For each patient, we recorded their clinical information such as sex, age, and BMI, their lifestyle information, such as smoking history and drinking history, their pathological result information such as *Helicobacter pylori* infection, atrophy, and intestinal metaplasia, and their traditional Chinese medicine phenotype information such as dry mouth, bitter taste, and gastric distention. A tongue coating sample was collected from each patient, and gastric fluid samples were extracted during the gastroscopy examination. The patient composition consisted of 18 males and 17 females, with an average age of 56 years old. Of these, 14 were *H. pylori-*positive patients and 21 were *H. pylori*-negative patients. The patients were divided into different disease developmental stages according to Correa’s gastric precancerous cascade on the basis of pathological examination results, and there were 16 cases of superficial gastritis, 7 cases of atrophic gastritis, 4 cases of intestinal metaplasia and 8 cases of dysplasia.

### Selection of samples

This research passed the ethical review of the medical ethics committee of China-Japan Friendship Hospital, and all the participants signed informed consent before enrollment. All the patients were from Chin-Japan Friendship Hospital; patients who were confirmed to have chronic gastritis by gastroscopy and pathological examination were enrolled in the study. Autoimmune gastritis patients were excluded. All pathological examination samples were from the gastric antrum, and all pathological examination results were recorded. *H. pylori* infection was determined by pathological examination. The inclusion standard was infirmed gastritis patients aged 18 to 80. Exclusion criteria were: 1. pregnant women, women trying to get pregnant, and lactating women; 2. patients with other diseases of the digestive system such as liver disease that could result in stomach pain; 3. patients with confirmed severe primary disease unrelated to chronic gastritis such as of the five sense organs, cardiovascular and cerebrovascular system, respiratory system, urinary system, digestive system, endocrine system and hemopoietic system; 4. mental disorder patients who could not express their feelings or receive investigation; and 5. patients who were treated with anti-*H. pylori* drugs, glucocorticoids and antibiotics within three months of enrolment.

### Extraction and preservation of gastric fluid and tongue coating samples

The tongue coating samples were collected the same day as gastroscopy examination, before gastroscopy examination and breakfast. Patients were required to fast after 10 pm the day before gastroscopy examination. Samples were collected using tongue coating swabs. First, three 1.5-ml Eppendorf (EP) tubes marked with 1, 2, and 3 were prepared, and 1 ml of phosphate buffered saline was added to each tube. Second, one tongue coating swab was used to scrape the tongue coating from the base to the tip of the tongue 30 times, while simultaneously turning the swab. Then, the swab was placed into EP tube 1, the swab was shaken to wash off the sample, and the samples were washed in tubes 2 and 3 in turn. The second swab was used to scrape the tongue coating the same way as stated above, and then the samples were washed in tubes 2 and 3 in turn. The third swab was used to scrape the tongue coating and wash the sample in tube 3. The three tubes of suspension were centrifuged at 2000×*g* for 2 min, and three tubes of supernatant, each containing 900 µl, or a total of 2.7 ml were collected into two new 1.5-ml EP tubes. Each new tube was centrifuged at 20,000×*g* for 10 min, the supernatant was discarded, 50 µl of precipitate was retained, and the tube was stored at − 80 °C.

Gastric fluid was collected during gastroscopy examination into a collector. Gastric fluid was moved into a 15-ml EP tube, and an equal amount of PBS was added. The mixture was divided into 1.5-ml EP tubes and centrifuged at 3000×*g* for 5 min. The supernatant was transferred to a new EP tube and centrifuged at 20,000×*g* for 15 min, and then the precipitate was stored at − 80 °C.

The microbial DNA of the tongue coating and the gastric fluid samples was extracted using an MO-BIO PowerSoil DNA Isolation Kit (MO-BIO Laboratories, Inc., Carlsbad, CA, USA) following the procedure in the instruction manual.

### Metagenomic sequencing

The microbial DNA was cut into segments with lengths of 400–500 bp using a Biouptor Pico nucleic acid interrupter, and the microbial genome DNA library was constructed by an ND604-VAHTS Universal DNA Library Prep Kit. Then, the DNA was sequenced using the Illumina 2500 sequencing platform with a reading length of PE125.

### Data preprocessing

SolexaQA [16] was used to perform quality control and quality filtering for raw data, and low-quality bases and sequences were removed using a threshold of 20. The DynamicTrim algorithm in SolexaQA kept the longest continuous subsequence satisfying the quality threshold. Sequences with lengths of more than 30 bp were retained after quality filtering.

High-quality data was mapped to the human genome (hg19, Genome Reference Consortium Human Reference 37) using SOAPAligner2 [17] to remove human sequences from the data. A threshold was set for the mapped length of 30, and the mapped ratio of 90. Thirty bases from the 5' end of the sequence were taken as the seed sequence, and the full-length sequence was aligned when the seed sequence matched successfully.

MetaPhlAn2 [18] was used to analyze the microbial species composition and abundance in the community. High quality microbial sequences were mapped to marker gene libraries, which could be classified at the species level of bacteria, archaea, eukaryotes and viruses. HUMAnN2 [19] was used to compute the microbial genes and pathways. HUMAnN2 first analyzed the microbial species composition in the community. Second, the sequences were aligned to the pan-genomic database ChocoPhlAn, and then the unmatched sequences were compared to the UniRef90 database using DIAMOND [20] software. Finally, the abundance information of genes and metabolic pathways were calculated.

### Statistical analyses

The correlated microbes between gastric fluid and tongue coating samples were tested by the Spearman correlation coefficient test followed by FDR correction. The Wilcoxon rank-sum test was used to compare the distance between the tongue coating and gastric fluid samples from the same patient and for the different patients.

## Discussion

In traditional Chinese medicine, the variation of the tongue coating is related to physical condition. However, the underlying mechanism of this relationship is unclear. This paper attempts to explain the scientific mechanism from the perspective of microorganisms. We detected the microbiome of the tongue coating and the gastric fluid using shotgun metagenomic sequencing, using gastritis patients as an example.

Evidence from many clinical studies indicates that variation in the tongue coating microbiome is associated with the occurrence and development of gastric disease [1, 2, 4, 21–23]. For a long time, it was thought that there were no microbes in the stomach, except in cases of *H. pylori* infection, until other microbes were found in later studies [8, 24, 25]. P. Sahay found *Campylobacter jejuni* in the gastric biopsy of a women by culturing, biochemical tests and PCR amplification [26]. Elias Hakalehto et al. found lactic acid bacteria in gastric biopsy samples [27]. Erik C. von Rosenvinge found that the stomach fluid microbiota was altered with changes in immune status, antibiotic medication and pH. The development of sequencing techniques allows the possibility of studying the microbiome in the stomach, and changes in the gastric microbiome have been suggested by many studies to have a strong correlation with the occurrence of diseases, especially stomach diseases [6, 9–12, 28–33].

Other studies have found both gastric juice and tongue coating microbiome variation under states of disease. Yubin Zhao profiled the gastric mucosa and tongue coating microbiome of chronic gastritis patients using 16S rRNA gene sequencing, and found that the number of bacterial interactions was greatly reduced in both the tongue coating and gastric microbiota of the *H. pylori*+/CagA+ samples [34]. However, the systematic relationship between the tongue coating and gastric juice microbiome has not been studied.

This paper constructed a microbiome atlas of tongue coatings and gastric juices using metagenomics. We found that both the tongue coating and gastric fluid microbiomes were dominated by Actinobacteria, Bacteroidetes, Firmicutes, Fusobacteria, and Proteobacteria, which is consistent with previous research [2, 9, 24], where the two sites were investigated separately. The systematic relationship between the microbiome of the tongue coating and the gastric juice was established for the first time, which provided a biological basis for studying the health status of the stomach from the perspective of the tongue coating microbiome.

In the theory of Traditional Chinese Medicine, tongue coating is produced by Qi of stomach, and the health state of the stomach could be inferred by observing the shape of the tongue coating, such as color, thickness, dryness, wetness, and so on. Our research found that there is a strong correlation between the tongue coating and the gastric fluid in terms of the composition and diversity of microbiome. On the one hand, microbes entering the mouth are carried to the stomach by swallowing and eating; on the other hand, microbes in the stomach can enter the mouth through reflux, burping, etc. It has been shown that microbiome in the stomach is correlated with the health status of the stomach. We speculate that metabolites produced by microbes accumulated in the tongue could cause changes in the morphology of the tongue coating. This may be one of the biological bases for inferring the health status of the stomach through tongue diagnosis in TCM.

This study found some species correlated between tongue coating and gastric fluid. We speculate that species that could be resistant to the low pH environment in the stomach may be shared in two locations. Some species were validated by other research. For example, *Porphyromonas gingivalis, Streptococcus salivarius, and Neisseria subflava*, which were correlated between tongue coating and gastric fluid, were shown to overcome changes in pH and survive in the stomach [35–37]. The biological reasons for these results need to be further studied. Microbiome of patients with different TCM phenotypes was analyzed, but no significant differences were found due to small sample size limitation. In further studies in larger sample size, this should be an important research direction.

As a critical microbe in the stomach [38–53], *H. pylori* could be detected by metagenomic sequencing in the gastric fluid of HP-positive patients. Furthermore, the abundances of Betaproteobacteria and Gammaproteobacteria, which belong to the same phylum as *H. pylori*, were found to be higher in the gastric fluid of HP-negative patients, which was similar to the research finding by Llorca [54]. Betaproteobacteria and Gammaproteobacteria were also shown to be increased in gastric adenocarcinoma patients [55]. From the perspective of genes, Betaproteobacteria and Gammaproteobacteria both have urease, flagella and chemotaxis genes, which are involved in the colonization of the gastric epithelium in *H. pylori *[56–58]. This gives us a hint that Betaproteobacteria and Gammaproteobacteria are related to the occurrence and development of gastritis. This may also explain why some people with not *H. pylori* infection are diagnosed with gastritis [59], while in some cases gastritis patients with *H. pylori* infection are not cured by eliminating *H. pylori*. We speculate that microbes belonging to the same phylum as *H. pylori* may play a similar role as *H. pylori* in gastritis patients. Further studies are needed to reveal the mechanism.

In future research on larger samples, taking pH value information into consideration in the study may reveal more specific results. At the sample processing stage, the Bead-beating step could be taken before DNA extraction to compare the results and get a more accurate assessment of the abundance and composition of the microbiome. Furthermore, more samples which including healthy cohort are needed to verify the reliability of the results, and confounding factors that could influence on the results should be verified.

## Conclusions

This study elucidates the health relationship between tongue coating and stomach from the perspective of microorganisms, which provide a scientific basis for tongue diagnosis. A better understanding of tongue diagnosis could lead to the development of non-invasive diagnosis and better treatment of gastric diseases.

## Data Availability

The datasets generated during the current study can be downloaded from Sequence Read Archive with Accession number: PRJNA755898 (https://dataview.ncbi.nlm.nih.gov/object/PRJNA755898?reviewer=506st0lut458fv9hc911lj3dke). The code during the analyses can be downloaded from GitHub (https://github.com/JiaxingCui/Tongue-coating-and-gastric-fluid-analysis).

## Abbreviations

TCM: Traditional Chinese medicine
KEGG: Kyoto Encyclopedia of Genes and Genomies
Kos: KEGG Orthologs
EP: Eppendorf
H. pylori: Helicobacter pylori
HP: Helicobacter pylori
